# Ozone protects cardiomyocytes against ischemia/reperfusion injury: Regulating the heat shock protein 70 (HSP70) expression through activating the JAK2/STAT3 Pathway

**DOI:** 10.1080/21655979.2021.1974760

**Published:** 2021-09-13

**Authors:** Shenglong Yu, Huizhuang Guo, Yi Luo, Hanwei Chen

**Affiliations:** a The first clinical college of Jinan University, Guangzhou, China; b Department of Cardiovascular, Panyu Central Hospital, (Cardiovascular Institute of Panyu District), Guangzhou, China; c Department of Radiology, Panyu Central Hospital, (Medical Imaging Institute of Panyu District), Guangzhou, China; d Department of Cardiovascular Medicine, First People’s Hospital, Guangzhou, China

**Keywords:** Ozone, ischemia/reperfusion injury, JAK2/STAT3, HSP70, myocardial infarction

## Abstract

Ischemia/reperfusion (I/R) injury causes complications in early coronary artery reperfusion for acute myocardial infarction (AMI). Ozone (O_3_) has been reported to be applied for protecting I/R injury, but its detailed mechanism remains unclear. Our study focused on the protective effect of O_3_ pretreatment on myocardial I/R injury and JAK2/STAT3 signaling and HSP70 regulation involving in the mediation. The rat hearts which were perfused and isolated as well as the cultured cardiomyocytes of neonatal rat were exposed to hypoxia/reoxygenation (H/R) and different concentrations of O_3_ followed by heat shock protein 70 (HSP70) siRNA treatment. The results showed O_3_ attenuated the suppression of cell viability induced by H/R and decreased the release of activity of creatine kinase (CK), lactate dehydrogenase (LDH) and apoptosis of cardiomyocytes *in vitro*. Moreover, O_3_ also activated the JAK2/STAT3 signaling, upregulated the expression of HSP70 both *in vitro* and *vivo*, and decreased the index of apoptosis of cardiomyocytes caused by I/R as well as myocardial infarct area *in vivo*. In addition, HSP70 siRNA and JAK2 inhibitor AG490 inhibited the cardioprotective effect of O_3_. And the expression of HSP70 increased by ozone was reduced by AG-490. In conclusion, our results demonstrated that ozone protects cardiomyocytes in I/R injury through regulation of the expression of HSP70 by activating the JAK2/STAT3 pathway.

## Introduction

Globally, acute myocardial infarction (AMI) is the most morbid and mortal disease worldwide, and coronary artery reperfusion in the early stage is the most effective and important treatment [1]. But, this can also result in paradoxical ischemia/reperfusion injury (IRI) [2], inducing some fatal reperfusion injury such as myocardial stunning, the phenomenon of no-reflow and arrhythmia caused by reperfusion [3]. However, IRI is a complex process involving oxidative stress, inflammation, intracellular Ca^2+^ overload, neurohumoral activation cell, death by apoptosis and necrosis [4,5].

Signaling pathway of transcription 3 (STAT3) is a signal transducer and activator that, along with Janus kinase 2 (JAK2), is widely participated in the body’s biological processes. And, it is a mechanism that can transmit signals from the cell surface to the nucleus through a stress response [6]. The JAK2/STAT3 pathway is an important string associated with regulation of cell proliferation, differentiation, apoptosis and inflammatory response [7]. Studies show that the JAK2/STAT3 signaling pathway is particularly participated in the prevention of myocardial IRI [8,9], especially by generating proteins for protection, including LOS and COX-2 through regulation of expression of Bcl-2/Bax [10].

As a member of the heat shock protein 70 (HSP70) superfamily, HSP70 is found in nearly every cellular compartment in eukaryotes [11]. It has an essential role in cellular protein metabolism due to its ability to promote nascent folding of polypeptides and remove denatured proteins [12]. HSP70 expression produces cells that are highly resistant to death caused by oxidative stress, tumor necrosis factor (TNF), heat, overexpression of caspase-3, UV radiation and several chemotherapeutic drugs [13–15]. Numerous reports found that overexpression of HSP70 enhances myocardial tolerance to IRI [16,17]. In addition, it is also allowed to activate HSP70 through the JAK/STAT pathway [18]. Xu [15] et al. demonstrated that blocking the JAK2/STAT3 signaling pathway downregulated HSP70 expression, thereby inhibiting Raji cell proliferation, inducing cell cycle arrest, and promoting oxidative stress and apoptosis. Guo et al. [19] found that matrine could protect cardiomyocytes from IRI by activating the JAK2/STAT3 pathway to upregulated HSP70 expression. Yu et al. [20] similarly showed that DL-3-n-butylphthalide protects H9c2 cardiomyocytes from ischemia/reperfusion injury by increasing the HSP70 expression through activation of the PI3K/AKT pathway.

Presently, clinicians use ozone (O_3_) to manage diseases related to infections, ischemia, and inflammation, as well as strokes and peripheral vascular disorders, peritonitis, bedsores in diabetic and non-diabetic patients, and other conditions [21]. Studies confirm that the relative risk of ischemic (infarct extension) and complications of arrhythmia can be reduced by ozone [22]. In rat models of renal and hepatic ischemia, ozone oxidative preconditioning protected against IRI [23,24]. Ozone administration also reduced reperfusion injury in isolated rat heart models [25]. Yet another study reported ozone exerted protective effects against liver IRI through regulation of HSP70 [26]. Based on the above studies, we speculate that ozone may also exert a protective effect against cardiac IRI by activating the JAK2/STAT3 pathway and upregulating the expression of HSP70. Therefore, we explored the mechanisms of HSP70 regulation and JAK2/STAT3 signaling pathway in the role of ozone protection in cardiac IRI through *in vivo* and *in vitro* experiments.

## Materials and methods

### Animal model of ischemia/reperfusion (I/R) injury

This study was authorized by the Ethics Committee on Animal Experiments, Jinan University (Guangzhou, China) and followed the principles of People’s Republic of China National Guidelines for Laboratory Animal Welfare (https://oacu.oir.nih.gov/animal research advisory committee guidelines).

Thirty male adult Sprague-Dawley (SD) rats, aged 7 weeks of (200–220 g), were acquired from Beijing Vital River Laboratory Animal Technology Co., Ltd. (Beijing, China). Raised in a light/dark circle for 12 h in an environment of controlling the temperature and humidity, the rats were supplied with regular food and water *ad libitum*. Ligation was performed for 30 min to induce I/R, and then the left anterior descending (LAD) coronary was reperfused for 2 h according to the previous studies [27,28]. Briefly, after being fully anesthetized with inhalation of 1.5–2% isoflurane, the hearts were exposed and the LAD coronary artery was ligated by 6–0 suture. Myocardial ischemia was confirmed once the saddleback-type ST segment elevation and a significant T wave increase was recorded by electrocardiogram. After occlusion for 30 min, the ligation was taken away and the LAD was reperfused for another 2 h. The sham operation group was performed the same surgical procedure but the LAD was not ligated.

### Animal experiment groups

Thirty rats were separated to five groups randomly (*n = 6* per group): sham operation group, I/R group, I/R + 50 μg/kg O_3_ group, I/R + 100 μg/kg O_3_ group and I/R + 100 μg/kg O_3_ + AG490 group. Rats in I/R+ O_3_ groups had continuous intraperitoneal injection of 50 μg/kg or 100 μg/kg O_3_ for 5 days before I/R treatment, and the rest of the groups were performed the same volume of air. Rats in the I/R + 100 μg/kg O_3_ + AG490 group received JAK2 inhibitor AG-490 injection before ozone treatment.

### Evaluation of LDH and CK-MB levels in serum

The LDH and CK levels in blood samples and supernatant in each group were determined by ELISA using commercially available kits (Nanjing Jiancheng Bioengineering Institute, Jiangsu, China) according to the manufacturer’s protocols. The blood samples (5 ml) were collected after reperfusion. Separated and stored the serum at −80°C until assayed.

### Myocardial infarct size measurement

The myocardial infarct size was assessed by staining with triphenyl tetrazolium chloride (TTC; Sigma‐Aldrich, USA) [29]. Heart tissues were isolated after the blood sample collection and the ventricles were cut to 5 slices, and then were kept at 37°C of 1% TTC for 10 min and fixed in 4% paraformaldehyde solution. The software of Image J (NIH, Bethesda, MD) could quantify the infarct area (white unstained by TTC) and non-infarcted area (red stained by TTC) and capture images afterward.

### Isolation and culture of rat cardiomyocytes

Cardiomyocytes were separated from the ventricles of SD rats (24 h Postnatal) of primary neonatal [30]. In short, the rats were anesthetized with 5% isoflurane. The thoracic cavity was opened under sterile conditions, and the heart was then removed and cut into small pieces. They were then digested by collagenase/dispase (Roche, Germany). Filter the cell suspension through a cell strainer to get rid of debris of larger tissue. After preplanting for 2 h, fibroblasts and endothelial cells were removed and non-adherent cells were collected. Subsequently, the cells were seeded in 6-well plates with DMEM (Thermo, USA) medium which contains 10% fetal bovine serum (FBS; Gibco, USA), and then incubated at 5% CO_2,_ 37°C for 72 h. We digested and seeded the cardiomyocytes into appropriate cell culture plates for the next step.

### Hypoxia/reoxygenation (HR) and cell treatment

The cardiomyocytes were subject to the following treatments: H/R treatment hypoxia (4 h, 5% CO_2_ and 1% O_2_/94% N_2_) and reoxygenation (6 h, 95% O_2_/5% CO_2_); ozone treatment (30 min) followed by H/R treatment; AG-490 treatment (2 μm, 1 h), ozone treatment and H/R treatment; HSP70 siRNA treatment, ozone treatment and H/R treatment.

### CCK8 assay

Cell viability was evaluated through analyzing Cell Counting Kit-8 (CCK-8). Cardiomyocytes were seeded in a 96-well plate. H/R injury and Ozone treatment were conducted like described above. Several concentrations of O_3_ (0, 5, 10, 20, 30, 40, 80 μg/ml) were used for reoxygenation and after 6 h, 10 μL CCK8 (Nanjing Jiancheng, China) was seeded to each of the well and kept in dark for 2 h at 37°C. A microplate reader (Thermo, USA) was used to measure the absorbance at 450 nm.

### Western blot assays

According to the protocol described above, Western blot analysis was performed [31]. Cardiomyocytes were collected and split by RIPA lysis buffer, which contained 1% phenylmethylsulfonyl fluoride. In order to eliminate the insoluble matters, the lysate was centrifuged at 12,000 g, 4°C, lasting for 15 min. By using a commercially available kit (Thermo, USA) and bicinchoninic acid assay, the concentration of protein was measured. Using 12% SDS-PAGE to separate the protein samples (20 μg) of each group, which were then blotted on polyvinylidene difluoride (PVDF) membrane (Merck Millipore, Germany). For blocking the nonspecific binding, the membranes were incubated for 2 h at room temperature in skimmed milk powder of 5% (w/v) in Tris-buffered saline which contained Tween-20 (TBST) of 0.05% (v/v). Thereafter, with the appropriate primary antibodies, the membranes were incubated at 4°C overnight. Information on antibodies is listed as follows: JAK2 (1:1000, ab108596, Abcam, UK); HSP70 (1:1000, ab2787, Abcam, UK); p-JAK2 (1:1000, ab32101, Abcam, UK); Bax (1:1000, ab32503, Abcam, UK); p-STAT3 (1:1000, ab76315, Abcam, UK); Caspase-3 (1:1000, ab184787, Abcam, UK); STAT3 (1:800, ab119352, Abcam, UK); Bcl-2 (1:800, ab196495, Abcam, UK), and β-actin (1:2000, ab179467, Abcam, UK). Washed for 30 min with TBST, the membranes were incubated with the proper secondary antibodies conjugated with HRP at 1:4,000 dilutions. The BioRad imaging system (Bio-Rad, USA) was then used to visualize the membranes with enhanced chemiluminescence.

### siRNA transfection

According to the instruction of manufacture, the reagent (Thermo Scientific) was transfected with TurboFect siRNA and the cardiomyocytes were transfected with control siRNA and *HSP70* targeted siRNA (GenePharma Co., Ltd, China). The specific siRNA information is manifested below: scrambled siRNA: 5ʹ-CCUCGUGCCGUUCCAUCAGGUAGUU-3ʹ (Sense) and 5ʹ-CUACCUGAUGGAACGGCACGAGGUU-3ʹ (Antisense). Cells were collected and analyzed by western blot to ensure the knockdown efficiency of HSP70 after 24 h.

### Real-time RT-PCR analysis

The total RNA from cardiomyocytes was extracted by TRIzol (Invitrogen, USA), and purified by using the RNeasy Mini Kit (Qiagen, Germany). By using Roche Lightcycler 480 Detection System, the specific products were amplified and detected. The difference in *HSP70* mRNA expression between groups was expressed using cycle time (Ct) values. The expression of *HSP70* in relation to *β-actin* was determined by 2^−ΔΔCt^ method. The primer sequences for *HSP70* gene are: GTGCGGCCTTAGTAGAGGTG (F) and GCTGGTGTCTGTGGCTGTTG (R).

### Statistics analysis

All data are showed as the mean ± standard deviation (SD) and analyzed by GraphPad Prism8.0 (GraphPad Prism Software, USA). Using one-way analysis of variance to perform statistical analyses, and followed by Tukey’s post hoc test. When *p* < 0.05, it was believed to be significant.

## Results

### *Ozone prevented cardiomyocytes injury induced by H/R* in vitro

**Figure 1.**
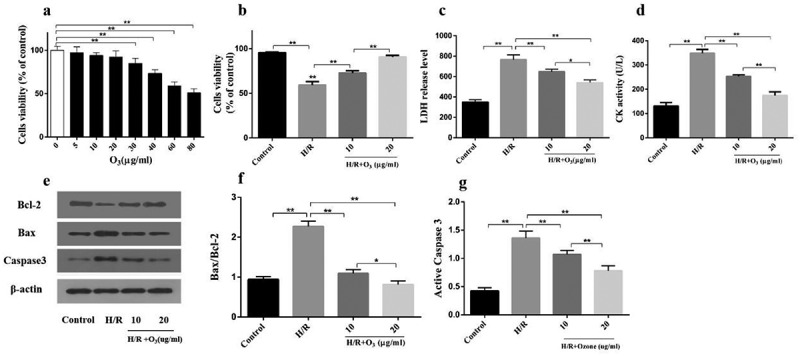
Ozone prevents cardiomyocytes from injury caused by H/R *in vitro*. (a) Treat various concentrations (0, 5, 10, 20, 30, 40, 60, 80 μg/ml) of ozone to the cardiomyocytes of primary rat and choose CCK-8 analysis to test cell viability. (b) Treat with 10 μg/ml or 20 μg/ml to cardiomyocytes after 4 hours of hypoxia, and then incubate in a normal incubator with oxygen for 6 hours to reoxygenate, and measure the viability of cell by CCK-8. (c) Release level of LDH. (d) CK activity. (e) analysis of Western blot about the levels of protein of Bax, cleaved caspase-3, Bcl-2 and caspase-3. (f) Quantification of Bax/Bcl-2 ratio. (g) Activity level of caspase-3. n = 3 *p < 0.05, **p < 0.01

Effects of various ozone concentrations on the viability of cardiomyocytes was first measured with CCK-8 assay (Figure 1(a)). Ozone ranging from 5 μg/ml to 20 μg/ml did not affect the viability of cardiomyocytes. Therefore, we used 10 μg/ml and 20 μg/ml to test the effect of ozone on protecting the cardiomyocytes injury caused by H/R. As manifested in Figure 1(b-d), the cell viability was significantly inhibited, and release of LDH as well as the CK activity were increased by H/R when comparing with the control group. But, the cell viability was notably increased, and the release of LDH as well as the CK activity were decreased by ozone in a manner of dose dependent when comparing with the H/R group. Ozone inhibition on cardiomyocyte apoptosis was further demonstrated by Western blotting (Figure 1(e)). H/R-induced Bax/Bcl-2 ratio (figure 1(f)) and the level of active caspase-3 (Figure 1(g)) were dramatically decreased by applying ozone in a manner of dose dependent.

### Ozone attenuated myocardial injury induced by I/R in rats

**Figure 2.**
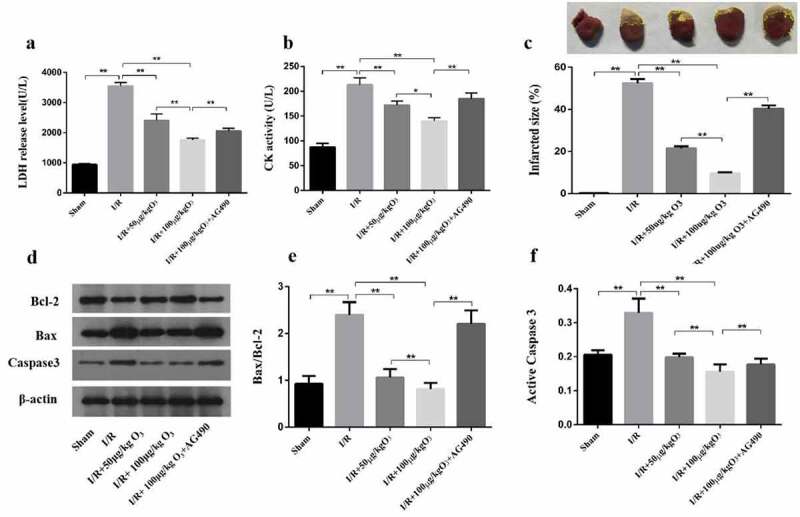
In animal models, ozone attenuated myocardial injury caused by I/R. (a) The LDH level of Serum. (b) CK-MB activity of Serum. (c) Representative photographs in TTC staining for heart tissues and the myocardial infarct size was calculated. (d) Levels of protein of Bax, caspase-3, cleaved caspase-3 and Bcl-2 were detected by Western blot. (e) Quantification of Bax/Bcl-2 ratio. (f) Activity level of caspase-3. n = 3 **p < 0.01

LDH level and CK activity were firstly detected in the serum while examining the effect of ozone in the myocardial injury induced by I/R in the rat model. As it manifested in Figure 2(a,b), the I/R group had a significant increase in LDH level and CK activity when compared with the Sham group, which was mitigated by ozone treatment in a dose-dependent manner. However, AG-490 treatment almost abolished the effect of ozone pretreatment. These results were consistent with TTC assay (Figure 2(c)). Induced by I/R, the infarcted size depended on dose and relieved by applying the ozone while AG-490 significantly attenuated the ozone protective function (Figure 2(d)). Next, the Bax/Bcl-2 ratio as well as level of protein of active caspase-3 were tested by Western blot (Figure 2(e)). The results manifested that IRI elevated the Bax/Bcl-2 ratio (figure 2(f)) as well as the active caspase-3 level (Figure 2(g)), while reduced by applying ozone in a manner of dose dependent, which suggested that ozone can reduce the myocardial injury caused by I/R in animal models.

### Ozone suppressed cardiomyocyte injury caused by H/R via activating the signaling of JAK2/STAT3

**Figure 3.**
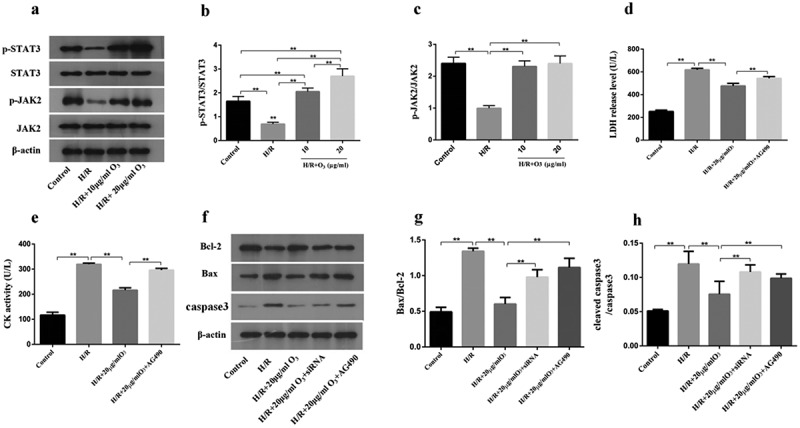
Ozone inhibited the injury of cardiomyocyte caused by H/R through activating the signaling of JAK2/STAT3. (a) Western blot to detect the protein levels of STAT3, p-JAK2, p-STAT3 and JAK2. (b) Quantification of p-STAT3/STAT3 ratios. (c) Quantification of p-JAK2/JAK2 ratios. 30 min before applying the H/R, cardiomyocytes, treated with JAK2 inhibitor AG-490 (2 mM), were incubated during the whole process of H/R. (d) The release level of LDH. (e) CK activity. (f) After assaying, the levels of expression of Bax, caspase-3, cleaved caspase-3 and Bcl-2 were determined by Western blot. (g) Quantification of Bax/Bcl-2 ratio. (h) Activity level of caspase-3. each group n = 3, **p < 0.01

The molecular mechanisms of ozone in myocardial injury caused by H/R were first determined *in vitro*. To confirm whether ozone protective functions were activated through the signaling pathway of JAK2/STAT3, we detected the protein levels of p-STAT3, STAT3, p-JAK2 and JAK2 as shown in Figure 3(a-c). H/R stimulation notably reduced the p-JAK2/JAK2 and p-STAT3/STAT3 ratio, which was regained by ozone treatment. 30 min before applying with H/R, the cells were pretreated with 2 mM AG-490, and incubated during the whole process of H/R. This was to confirm whether the ozone’s protection against injury to cardiomyocyte caused by H/R was functioned or not through activating the signaling of JAK2/STAT3. It showed that AG-490 had reversed the LDH level and CK activity of 20 μg/ml of ozone (Figure 3(d-e)), and the active level of caspase-3 and Bax/Bcl-2 ratio (figure 3(f-h)). Taken together, the data indicated that the cardiomyocyte injury caused by H/R was inhibited by ozone through activating the signaling of JAK2/STAT3.

### The effect of ozone mediated by HSP70 to cardiomyocytes injury caused by H/R

**Figure 4.**
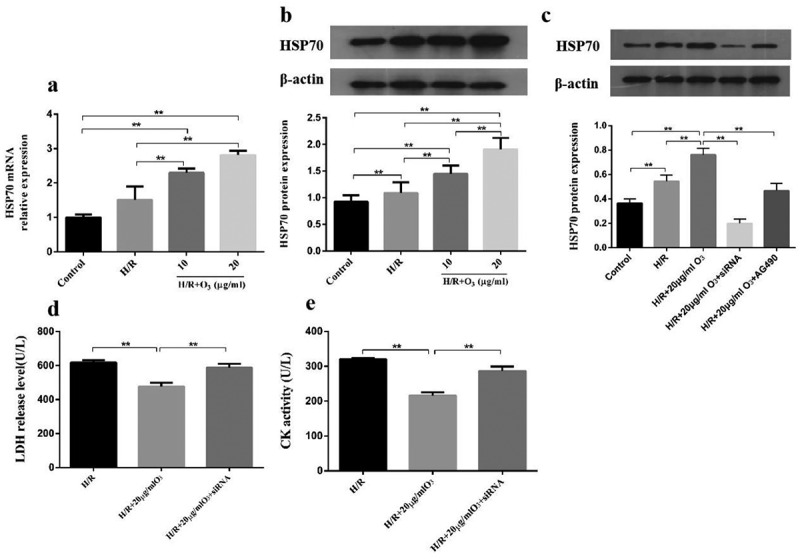
The effect of ozone mediated by HSP70 to Cardiomyocytes Injury induced by I/R. (a) The relative expression of HSP70 mRNA was detected by RT-PCR. (b) The protein level of HSP70 was assayed by Western blot. (c) Cardiomyocytes were transfected by *HSP70* siRNA, control or pretreated with AG-490 followed by 20 μg/ml ozone treatment and I/R treatment. HSP70 protein level was determined. LDH release level (d) and CK activity (e) in Cardiomyocytes were detected. n = 3 per group, **p < 0.01

Next, we studied the HSP70 function in cardiomyocytes protection during myocardial injury caused by H/R. We determined the protein levels and mRNA of HSP70 in cardiomyocytes. Compared with the control group, the protein levels and mRNA of HSP70 were elevated due to H/R; But ozone dose-dependently upregulated the HSP70 expression (Figure 4(a-b)). To further understand the ozone functions via upregulating the HSP70 expression, which had a similar function as AG-490 on the cardiomyocytes, we used *HSP70* siRNA 24 h before the treatment with H/R to abolish HSP70 expression in cardiomyocytes. And we found that *HSP70* siRNA had a similar function as AG-490 on the cardiomyocytes (Figure 4(c)). The results indicated that the effects of ozone to the CK activity and LDH level were attenuated by *HSP70* siRNA (Figure 4(d-e)).

### *Ozone attenuated myocardial injury induced by I/R via upregulating HSP70 and activating JAK2/STAT3* in vivo

**Figure 5.**
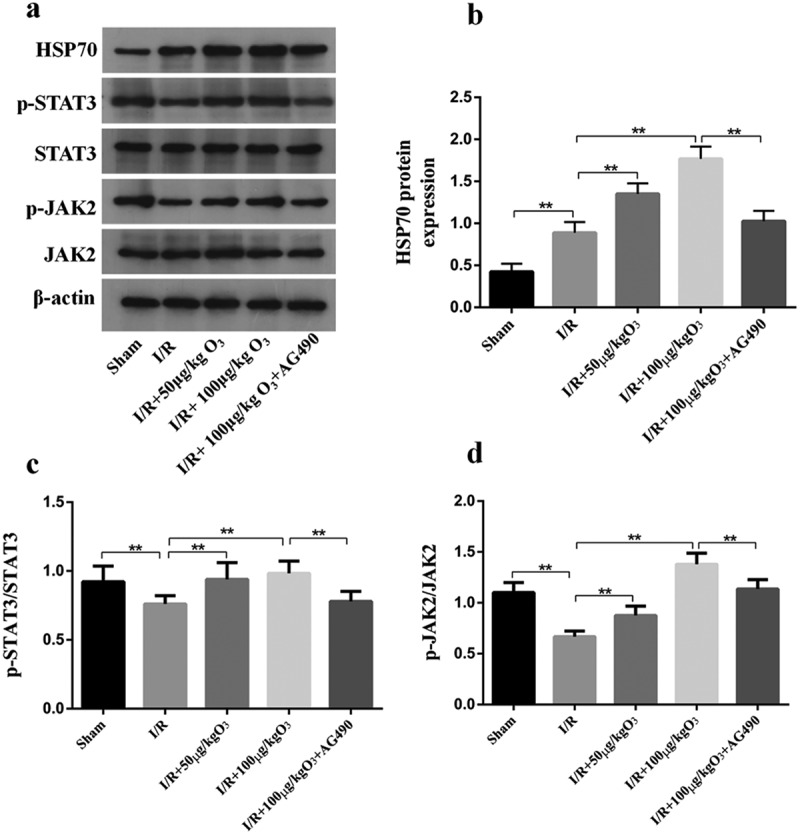
Ozone alleviated myocardial injury caused by I/R through up-regulating HSP70 and activating JAK2/STAT3 *in vivo*. (a) Analyzed the levels of protein of JAK2, p-STAT3, p-JAK2, HSP70 and STAT3 in I/R animal models. (b–d) Quantification of HSP70 level, as well as p-STAT3/STAT3 and p-JAK2/JAK2 ratios. n = 6, **p < 0.01

We investigated the roles of JAK2/STAT2 signaling in ozone pretreatment to cardiomyocytes *in vivo* and cleared the levels of p-JAK2, JAK2, p-STAT3, STAT3 by Western blot. As manifested in Figure 5(a), when compared with the Sham group, I/R injury led to a significant decrease in the ratio of pJAK2/JAK2 and pSTAT3/STAT3, as well as an increase in the expression of HSP70, which could be reversed by pretreating with the AG-490 (Figure 5(b-d)). We also found that rats exposed to AG-490 before I/R treatment and ozone therapy had decreased HSP70 levels. These results are consistent with those *in vivo*. In general, these results indicate ozone protects cardiomyocytes from injury caused by I/R through activating the pathway of JAK2/STAT3 and upregulating the HSP70.

## Discussion

Myocardial IRI is caused by a direct result of blood flow restoration to the ischemic tissue and can lead to cell death and added cardiac dysfunction [32]. Ozone administration is used in treatment for conditions associated with the tolerance to ischemia-induced damage [33], also called ozone oxidative preconditioning (OOP) [34]. However, ozone is a potential toxic substance, and continued exposure to high levels of ozone might increase the produce of reactive oxygen and inflammatory mediators [35]. We tested the therapeutically nontoxic ozone concentrations of 10–80 μg/ml *in vitro* and found that the ozone concentration of less than 30 μg/ml did not affect cardiomyocytes viability. Consequently, we chose 10 μg/ml and 20 μg/ml ozone for the following experiments *in vitro. In vivo*, we selected an ozone dose of 100 μg/kg as described in a previous study [36]. OOP provides protective effects against apoptosis, inflammation and oxidative stress in IRI [37,38]. In the present study, we found that ozone treatment reduced the area of I/R-induced myocardial infarction in rats. In addition, ozone significantly reduced CK activity and LDH release in serum of I/R rats and H/R-induced cardiomyocytes, and protected against myocardial injury by inhibiting active caspase-3 and Bax protein expression. These results are consistent with the findings of Chen et al. [39]. They found that interference with lncRNA MIAT reduced H/R-induced LDH release from cardiomyocytes, I/R-induced myocardial infarct size, and inhibited the expression of apoptotic proteins.

JAKs and STATs families of protein transduce extracellular signals into nucleus and activate the transcription of target genes [40]. STAT proteins are substrates of JAKs and can be phosphorylated to transform into an activated form whereby p-STATs translocate to the nucleus and transactivate responsive genes [40]. The JAK2/STAT3 signaling pathway regulates the expression of genes involved in cell-cycle progression, cell survival, cell proliferation, and angiogenesis [41]. JAK2/STAT3 signaling is important for myocardial protection. In the LAD ligation of a rat model, as early as 5 minutes after myocardial infarction, we observed JAK2, STAT1, STAT3, STAT5a, and STAT6 phosphorylation [42]. We also found that scutellarin modulates IRI-induced oxidative stress and apoptosis by enhancing JAK2/STAT3 pro-survival signaling [43]. Several genes related to apoptosis including Bcl-xl and Bcl-2, were classified as STAT3’s target genes [44,45]. Shinji Negoro et al. found that in rats AMI with coronary ligation and AG-490 treatment, apoptotic cells were notably increased, while pSTAT3 was significantly inhibited. Meanwhile, the expression of activity of caspase-3 and Bax in the myocardium were increased notably, which indicated that in AMI myocardium, the JAK2/STAT3 pathway, acting as a pivotal role in signaling of cytoprotective, was activated [46]. Considering the importance of JAK-STAT3 in preconditioning and cardioprotection, it was sufficient for activated JAK2/STAT3 to protect the myocardium from apoptosis. In this study, ozone showed a myocardial protective function in IRI, which led to a reduced number of apoptotic cardiomyocytes, a smaller myocardial infarct size and a decrease in LDH release and CK activity. In this process, ozone treatment could restore the reduced ratios of p-STAT3/STAT3 and p-JAK2/JAK2 caused by IRI, with up-regulating the expression of the anti-apoptotic protein Bcl2, down-regulating the expression of the pro-apoptotic protein Bax, as well as the Caspase-3. JAK2 inhibitor AG-490 reverses the above results. All data indicate cardioprotection was induced by ozone through activating the signaling pathway of JAK2/STAT3.

HSP70, a conserved protein, is widely expressed and related to the cytoprotection fighting against stresses [47]. It contributes to cardioprotection in IRI by suppressing reactive oxygen species generation [48], inhibiting cell apoptosis [49], preventing the process of cell death by influencing the activation of caspase-3 and caspase-7, or even further downstream events of caspase-3 and caspase-7 activation [50]. Our study showed that ozone can significantly increase the expression of HSP70 in a manner of dose dependent, which can be inhibited by AG-490. Also, HSP70 siRNA could inhibit HSP70 expression and attenuate the effects of ozone to the release of LDH as well as CK activity, demonstrating that ozone protected cardiomyocytes from IRI by upregulating the HSP70 expression.

## Conclusion

In summary, we studied the relationship between the expression of HSP70 and JAK2/STAT3 in the ozone treatment of cardiomyocytes IRI. The results indicate that ozone alleviates myocardial IRI by activating the signaling pathway of JAK2/STAT3 and upregulating the expression of HSP70 as well.

## Highlights

- Ozone attenuates the suppression of cell viability induced by hypoxia/reoxygenation (H/R).
- Ozone decreases the release of activity of CK, LDH and apoptosis of cardiomyocytes.
- Ozone protects cardiomyocytes in I/R injury through regulation of the expression of HSP70 by activating the JAK2/STAT3 pathway.

## Abbreviations

Acute myocardial infarction: AMI

Cell Counting Kit-8: CCK8

Creatine kinase: CK

Heat shock protein 70: HSP70

Hypoxia/reoxygenation: H/R

Ischemia/reperfusion: I/R

Janus kinase 2: JAK2

Lactate dehydrogenase: LDH

Left anterior descending: LAD

Signal transduction and activator of transcription 3: STAT3

Triphenyltetrazolium chloride: TTC
